# The inhibition role of miR-22 in hepatocellular carcinoma cell migration and invasion via targeting CD147

**DOI:** 10.1186/s12935-016-0380-8

**Published:** 2017-02-02

**Authors:** Ling-Juan Luo, Li-Ping Zhang, Chun-Yan Duan, Bei Wang, Na-Na He, Patima Abulimiti, Yan Lin

**Affiliations:** 1 The Second Department of Oncology, Affiliated Traditional Chinese Medicine Hospital, Xinjiang Medical University, Urumqi, 830000 People’s Republic of China; 2 The First Department of Oncology, Affiliated Traditional Chinese Medicine Hospital, Xinjiang Medical University, No. 116, Huanghe Road, Urumqi, 830000 People’s Republic of China

**Keywords:** miR-22, Hepatocellular carcinoma, Invasion, Migration, CD147

## Abstract

**Background:**

Recently, miR-22 is identified as a tumor-suppressing microRNA in many human cancers. CD147 is a novel cancer-associated biomarker that plays an important role in the invasion and metastasis of malignant tumor. However, the involvement of miR-22 in CD147 regulation and hepatocellular carcinoma (HCC) progression and metastasis has not been investigated.

**Methods:**

We measured miR-22 expression level in 34 paired of HCC and matched normal tissues, HCC cell lines by real-time quantitative RT-PCR. Invasion assay, MTT proliferation assay and wound-healing assay were performed to test the invasion and proliferation of HCC cell after overexpression of miR-22. The effect of miR-22 on HCC in vivo was validated by murine xenograft model. The relationship of miR-22 and its target gene CD147 was also investigated.

**Results:**

We found that the expression of miR-22 in HCC tissues and cell lines were much lower than that in normal control, respectively. The expression of miR-22 was inversely correlated with HCC metastatic ability. Moreover, overexpression of miR-22 could significantly inhibit the HCC cell proliferation, migration and invasion in vitro and decrease HCC tumor growth in vivo. Finally, we found that miR-22 interacted with CD147 and decreased its expression, via a specific target site within the CD147 3′UTR by luciferase reporter assay. The expression of CD147 was inversely correlated with miR-22 expression in HCC tissues.

**Conclusion:**

Our results suggested that miR-22 was downexpressed in HCC and inhibited HCC cell proliferation, migration and invasion through downregulating cancer-associated gene CD147 which may provide a new bio-target for HCC therapy.

## Background

Hepatocellular carcinoma (HCC) has become one of the most frequently occurring cancers, and is considered to be highly lethal, accounting for approximately one-third of cancer-related deaths worldwide [1, 2]. Despite advances in the understanding of the molecular mechanisms underlying HCC and improved treatments for HCC, the overall survival time is still limited [3].

Current studies have shown that microRNAs (miRNAs) can act as activators or inhibitors of tumor metastasis by targeting multiple signaling pathways involved in metastasis [4–6]. miR-22 is a 22-nt non-coding RNA and originally identified in HeLa cells as a tumor-suppressing miRNA. Subsequently, miR-22 was identified to be ubiquitously expressed in a variety of tissues [7]. Recently, several targets of miR-22 were reported to mediate its tumorsuppressive effect, such as tumor-suppressive PTEN, Max genes, p21, Sp1, CD147 and oncogene c-myc expression, etc. [7–11].

CD147 which is a 58-kDa transmembrane glycoprotein belonging to the immunoglobulin superfamily [12, 13] is highly expressed in many tumors, including breast cancer, lymphoma, oral squamous cell carcinoma, glioma, melanoma, lung, bladder, liver and kidney carcinomas [13–15]. It has been demonstrated that CD147 contributes significantly to tumor growth, metastasis and angiogenesis through stimulating the production of hyaluronan, multiple matrix metalloproteinases (MMPs) and vascular endothelial growth factor A (VEGF-A) [16]. It has been reported that miR-22 inhibits cell migration and invasion through targeting CD147 in breast cancer [11]. So we want to explore whether there exists this relationship of miR-22 and CD147 in the HCC progression.

In the current study, we validate the differential expression of miR-22 in HCC and investigated the function of miR-22 in migration and invasion of HCC cells. Furthermore, we identified CD147 as a target gene for miR-22 to regulate the invasion and metastasis of HCC cells in vitro. miR-22 might act as a tumor suppressor and serve as a potential therapeutic target in HCC. To the best of our knowledge, this is the first study to examine the regulation mechanism of miR-22 and CD147 in HCC migration and invasion.

## Methods

### HCC tissue specimens and immunohistochemical analysis

Thirty-four paired tissue specimens of HCC and adjacent non-tumor (ANT) tissues were obtained from Department of Hepatobiliary Surgery, Affiliated Traditional Chinese Medicine Hospital, Xinjiang Medical University. Informed consent was obtained from each patient. The thirty-four HCC tissues were divided into two groups: metastatic HCC tissues (patients with lung metastasis or portal vein cancer embolus, n = 20) and non-metastatic HCC tissues (n = 14). All of the tissues were obtained at the time of surgery and immediately stored in liquid nitrogen until use.

Immunohistochemistry was performed using Histostain-SP kits (Invitrogen, Carlsbad, CA, USA) according to the manufacturer’s instructions. Antibodies were purchased from Santa Cruz Biotechnology (Santa Cruz, CA, USA). Immunopositivity was independently evaluated by two pathologists. Expression of protein was evaluated as described previously [17, 18].

### Cell lines and culture conditions

The following cell lines were used in this study: human normal liver cell QZG [19], QSG-7701 [20] and HL-7702 [21]; human hepatocellular carcinoma cell lines: HepG2, BEL-7402, FHCC-98, SMMC-7721, HCC-9724, MHCC-97H (HCC cells with high metastatic potential) and MHCC-97L (HCC cells with low metastatic potential) [22]. All cell lines were purchased from Shanghai Institute for Biological Sciences (Shanghai, China). All cell lines were routinely cultured in RPMI-1640 medium (Hyclone Laboratories, Logan, UT) supplemented with 10% fetal calf serum (Gibco BRL, Rockville, MD, USA) at 37 °C in a humidified atmosphere of 5% CO_2_.

### RNA extraction and real-time quantitative RT-PCR

Total RNA was isolated from tissues and cell lines by using TRIzol reagent (Invitrogen, Carlsbad, CA, USA) according to the manufacturer’s instructions. Reverse transcription was performed using the Prime-Script RT reagent kit (TaKaRa, Otsu, Japan). The glyceraldehyde-3-phosphate dehydrogenase (GAPDH) mRNA and U6 small nuclear RNA were used as internal controls, respectively. All of the reactions were run in triplicate. Forward and reverse primers for miR-22, u6 snRNA, were 5′-AAGCTGCCAGTTGAAGAACTGTA-3′ and Universal Primer (Qiagen, Hilden, Germany), 5′-CTCGCTTCGGCAGCACA-3′ and 5′-AACGCTTCACGAATTTGCGT-3′, respectively. The primers for CD147 and GAPDH mRNA were 5′-TCGCGCTGCTGGGCACC-3′ and 5′-TGGCGCTGTCATTCAAGGA-3′, 5′-GGTCGGAGTCAACGGATTTG-3′ and 5′-ATGAGCCCCAGCCTTCTCCAT-3′, respectively. The relative expression levels were evaluated using the *ΔΔ*Ct method [15, 23].

### Plasmid construction and luciferase activity assay

The miR-22 overexpression vector was constructed according to previous [24] named as pcDNA3.1-miR-22. The full-length and mutant construct of CD147 3′-UTR were amplified [11]. Pooled multiple siRNAs targeting CD147 [25] were synthesized by Genepharma (Shanghai, China). All constructs were further confirmed by sequencing. Cell transfection and dual luciferase reporter assay were performed as described previously [26].

### Cell proliferation assay

Cells were plated in sixduplicate in 96-well plates (2 × 10^3^ per well) in 100 μL complete medium and allowed to attach overnight. 3-(4,5-dimethyl-2-thiazolyl)-2,5-diphenyl-2H-tetrazolium bromide (MTT) (20 μL at 5 mg/mL; Sigma, St. Louis, MO) was added every 24 h and incubated for 4 h. The supernatant was discarded, the precipitate was dissolved in 200 μL dimethyl sulfoxide (DMSO), and plates were read with a microplate reader at 570 nm [27].

### Wound-healing assay

1 × 10^6^ cells were seeded in six-well plates, cultured overnight, and transfected with miR-22 overexpression vector or NC, respectively. When the culture had reached nearly 90% confluency, the cell layer was scratched with a sterile plastic tip and then washed with culture medium twice and cultured again for up to 48 or 72 h with serum-reduced medium containing 1% FBS. At different time points, photographic images of the plates were acquired under a microscope and the data were summarized based on sextuple assays for each experiment.

### In vitro invasion and migration assay

MilliCell (12 mm diameter with 8 µm pores) chambers (Millipore, Bedford, MA, USA) were pre-coated with Matrigel (BD, Bedford, MA, USA) on the upper side. A total of 1 × 10^5^ serum-starved HCC cells were added to the upper compartment in medium supplemented with 0.1% serum, and the chambers were placed into 24-well plates with medium containing 10% serum. After 24 h at 37 °C, invaded cells on the lower membrane surface were fixed and stained with 0.1% crystal violet. Invasive activity was quantified by counting nine high-power fields (HPFs, 400×) per chamber. Mean values were obtained from at least three individual chambers for each experimental point per assay. The migration assay is the same with invasion assay excepting no matrigel was used and the permeating time for cells was 12 h.

### In vivo proliferation assay

BALB/c nude mice at 4–6 weeks of age were provided by the Laboratory Animal Research Center of Xinjiang Medical University, and the animal study was reviewed and approved by the Xinjiang Medical University Animal Care and Use Committee. A total of 1 × 10^7^ MHCC-97H cells stably expressing miR-22 were injected subcutaneously into nude mice. 60 days after injection, the mice were killed. Tumor volume was determined using direct measurement and calculated using the formula length × width^2^/2.

### Western blot analysis

Cell samples were lysed with RIPA buffer (Beyotime, China). Equal amounts (10 μg) of total protein were loaded, and then subsequently immunoblotted with the primary antibodies, including anti-CD147 and tubulin monoclonal antibodies (Santa Cruz, CA, USA). Proteins were detected using the Amersham enhanced chemiluminescence system (Pierce, Rockford, IL, USA) according to the manufacturer’s instructions.

### Statistical analysis

All statistical analyses were performed using the SPSS 16.0 statistical software package (SPSS, Chicago, IL, USA). The significance of the data was determined using Student’s *t* test. All the statistical tests were two-sided, and a *P* < 0.05 was considered significant.

## Results

### The expression of miR-22 is downexpressed in HCC tissues and cell lines

**Fig. 1:**
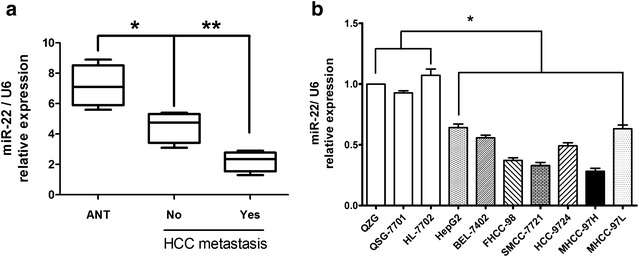
miR-22 is down-expressed in HCC tissues and cell lines. **a** Analysis of the expression pattern of miR-22 in non-metastasis tumors and metastasis tumors of HCC correlated with adjacent non-tumor tissues using real-time RT-PCR. **b** The relative levels of miR-22 in the seven HCC and three normal liver cell lines. Student’s *t* test was used to analyze the significant differences, *P < 0.05, **P < 0.01

To identify the expression of miR-22 in the HCC, thirty-four paired of HCC and normal tissues were measured by real time RT-PCR. As shown in Fig. 1a, miR-22 expression levels were downexpressed in HCC tissues compared with ANT tissues (*P* < 0.05). Furthermore, the expression of miR-22 in metastatic HCC tissues was much lower than in no metastatic HCC tissues (Fig. 1a) which indicated that the miR-22 expression was negatively correlated with the HCC metastatic ability.

The miR-22 expression in HCC and normal liver cell lines were also detected. We performed real-time RT-PCR on a panel of seven HCC and three normal liver cell lines. And miR-22 expression of each cell line was compared to the average expression level of miR-22 of three normal liver cell lines. As shown in Fig. 1b, miR-22 levels of all cell lines were lower than that of normal liver cell lines. miR-22 expression in MHCC-97H, FHCC-98 and SMMC-7721 cells was relatively low. In contrast, expression levels of miR-22 in HepG2 and MHCC-97L cells were relatively high. The lower expression of miR-22 in HCC cells with low metastatic potential suggested a causal role for miR-22 in the migration and invasion of HCC cell lines.

### miR-22 inhibits HCC cell proliferation, migration and invasion in vitro and decreases HCC tumor growth in vivo

**Fig. 2:**
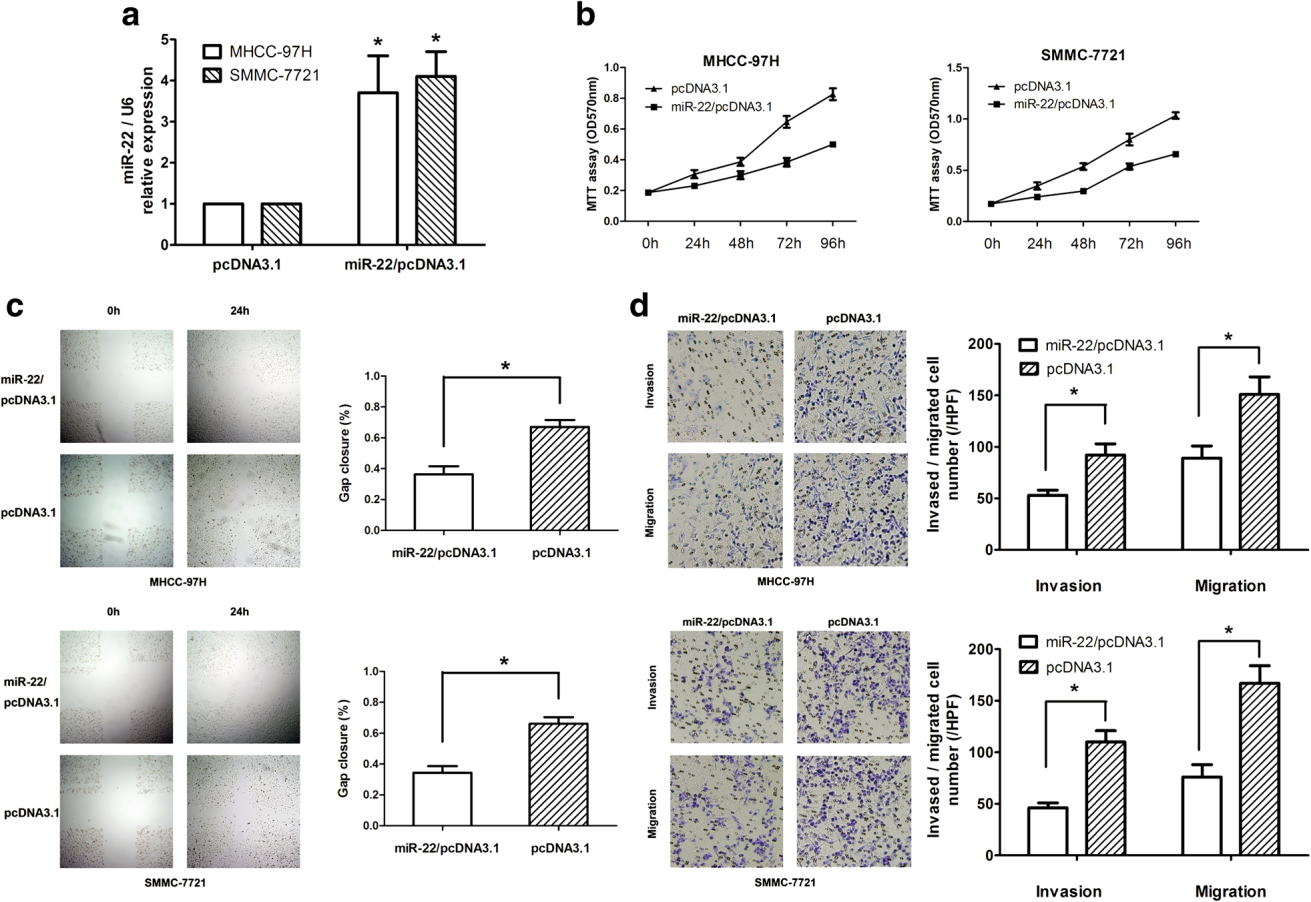
Overexpression of miR-22 inhibited HCC cell proliferation and metastasis in vitro. **a** Transfection of miR-22 overexpression vector to MHCC-97H and SMMC-7721 cells increases the expression of miR-22 detected by real-time quantitative RT-PCR. **b** Cell proliferation of these cells transfected as in **a** was measured at the indicated time periods using the MTT assay. **c** Overexpression of miR-22 presented a slower closing of scratch wound, compared with pcDNA3.1 control, at 48 h after transfection in MHCC-97H and SMMC-7721 cells. **d** Migration and invasion potential in both cell lines treated as above were measured by Transwell assays and were expressed as relative cell numbers. *Scale bars*, 50 μm. *Scale bars*, 50 μm. *P < 0.05

To investigate whether miR-22 regulates HCC cell migration and invasion, we selected MHCC-97H cells and SMMC-7721 which show strong migration and invasion potential for further study. First, we performed gain-of-function analysis and transfected miR-22 overexpression vector into MHCC-97H and SMMC-7721 cells to increase miR-22 expression. As expected, transfection of miR-22 overexpression vector resulted in substantial increase of miR-22 expression compared with pcDNA3.1 control transfected cells (Fig. 2a).

Then, we performed MTT cell proliferation assays with transfected miR-22 overexpression in MHCC-97H and SMMC-7721 cells. Our results showed that overexpression of miR-22 could significantly suppress HCC cell proliferation (*P* < 0.05, Fig. 2b). Next, the wound-healing assay showed that HCC cells with miR-22 overexpression presented a slower closing of scratch wound, compared with the negative controls (*P* < 0.05, Fig. 2c). Moreover, the cell migration and invasion assay showed that overexpression of miR-22 resulted in reduced migration rate and invasion rate of MHCC-97H and SMMC-7721 cells compared with the control (Fig. 2d). Our results indicate that miR-22 worked as a tumor suppressor microRNA and contributed to inhibit HCC cells migration and invasion in vitro.

**Fig. 3:**
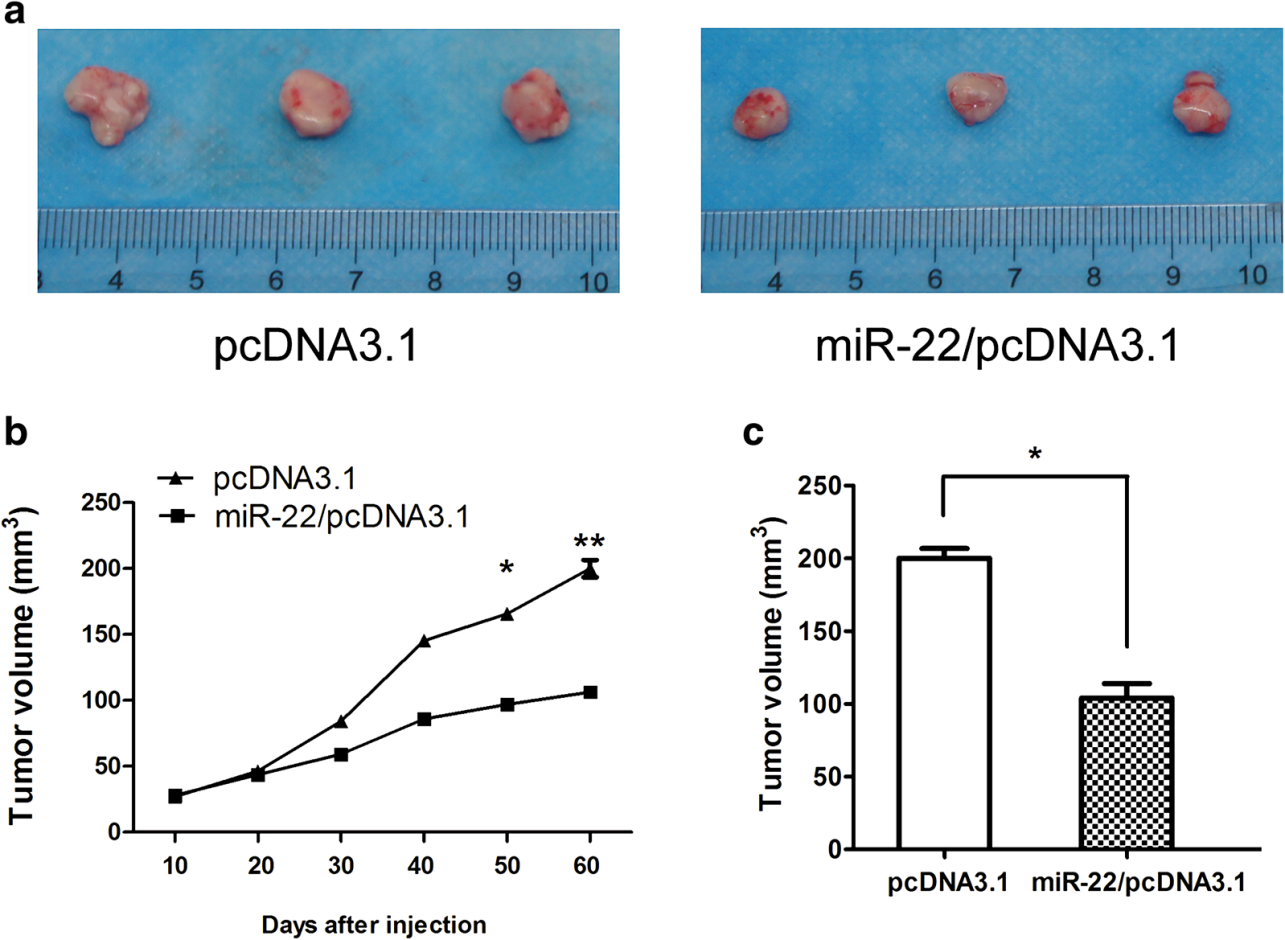
miR-22 decreases HCC growth in vivo. **a** Representative figures of tumors in negative control and miR-22 overexpression groups. **b** Determination of tumor volumes at different time points. **c** After the final measure, the mice were sacrificed, and the tumors were excised. Tumor volume was measured and calculated using the formula length × width^2^/2. Student’s t test was used to analyze the significant differences

We next asked whether miR-22 could inhibit HCC development in vivo. Using HCC tumor models, the control mice showed the apparent presence of primary tumor, whereas those injected with miR-22 expression vector decreased the volume and weight of tumors during the same observation period (Fig. 3). Judging from data between the controls and miR-22-treated groups at the points of the experiment, miR-22 treatment resulted in a mean of decreasing in tumor growth.

### miR-22 negatively regulates CD147 gene expression

**Fig. 4:**
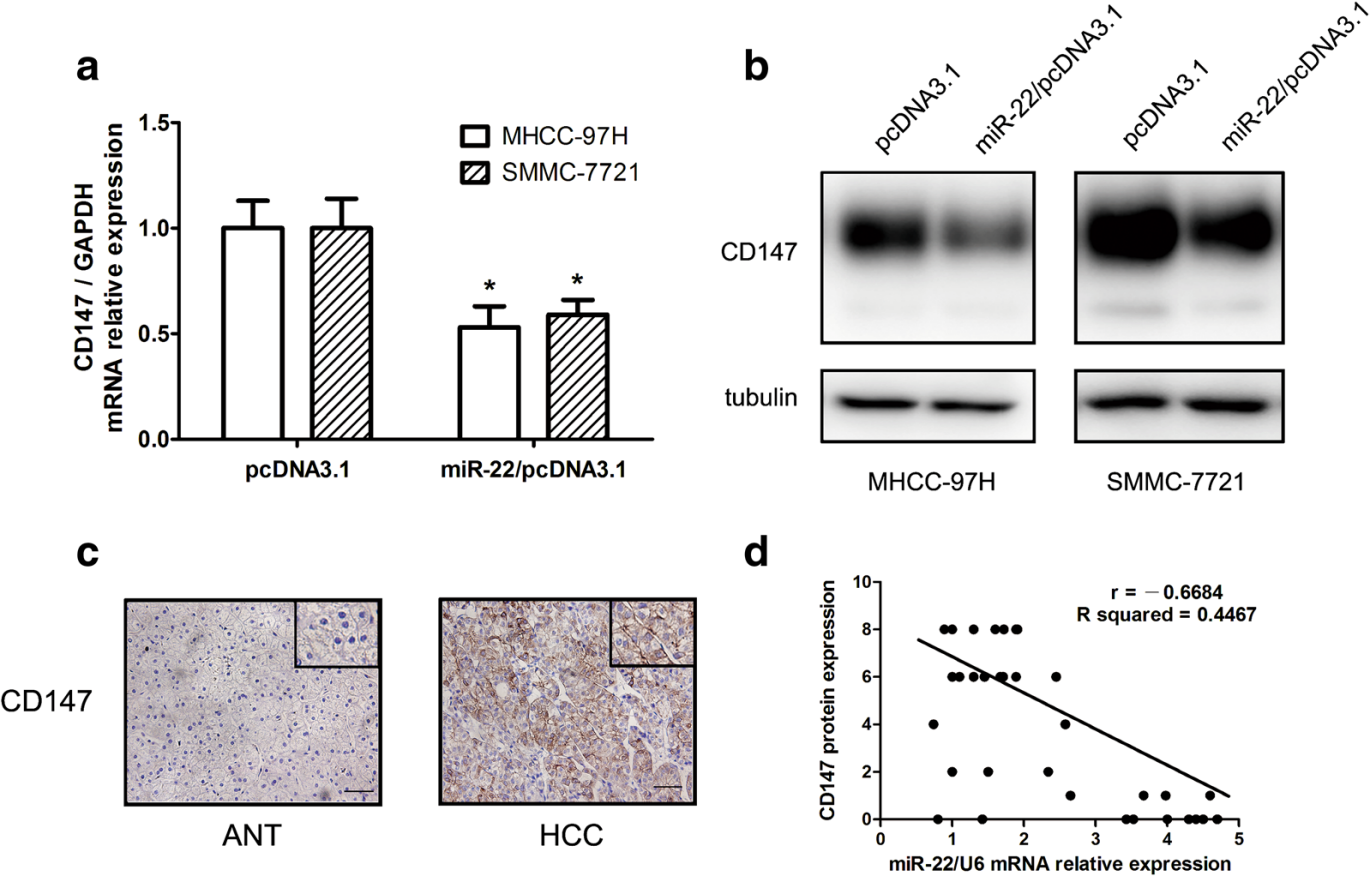
miR-22 negatively regulates CD147 gene expression. miR-22 inhibited the expression of CD147 at the mRNA level **a** and the protein level **b** in MHCC-97H and SMMC-7721 cells. **c** IHC analysis of CD147 protein expression in HCC and paired adjacent normal tissues. Pictures of representative areas are presented at different staining intensities (weak and strong) in ANT and tumor tissues. *Scale bars*, 50 μm. **d** Analysis for correlation of miR-22 mRNA and CD147 protein expression in HCC tissues

It has been reported that miR-22 could regulate CD147 expression in breast cancer [11]. So we want to explore whether there exists this relationship of miR-22 and CD147 in the HCC progression. To confirm that CD147 is a target gene for miR-22, real time RT-PCR and western blot analysis were used to detect the expression of CD147 after transfected with miR-22 overexpression vector in MHCC-97H and SMMC-7721. The expression of CD147 was significantly down-regulated after over expression of miR-22 at the mRNA level (Fig. 4a) and the protein level (Fig. 4b) compared with negative control, respectively.

Furthermore, we also detected the CD147 expression in thirty-four pairs of HCC and normal tissues by immunohistochemistry. CD147 was predominantly localized to the cytoplasm and membrane and the positive expression rate of CD147 was much higher in HCC (24/34) than that in ANT (11/34) (Fig. 4c). Correlation analysis indicated that there was a significant inverse correlation between the miR-22 mRNA and CD147 protein expression with a correlation coefficient (*r*) = −0.6684 and *R* ^*2*^ = 0.4467 (Fig. 4d). This correlation indicates that miR-22 could negatively regulateCD147 expression in HCC tissues. Taken together, our results suggest that miR-22 negatively regulates CD147 gene expression and that CD147 is a potential target gene of miR-22.

### CD147 is a direct target of miR-22

**Fig. 5:**
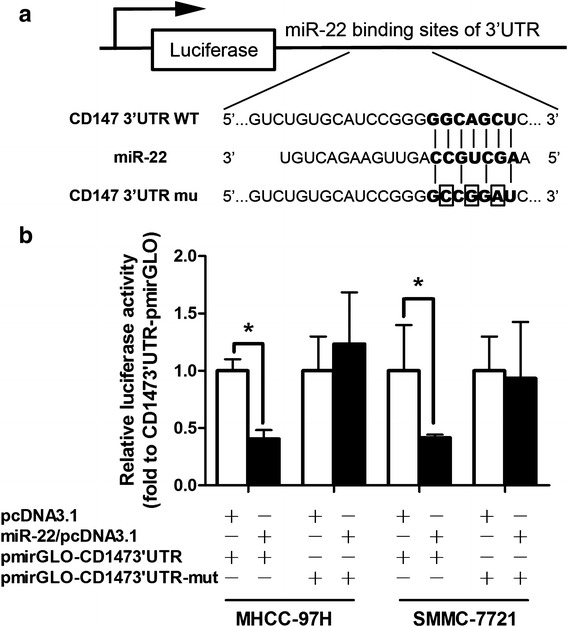
The CD147 3′UTR is a target of miR-22. **a** Diagram of the luciferase reporter plasmids: plasmid with the full length CD147 3′UTR insert and plasmid with a mutant CD147 3′UTR which carried a substitution of four nucleotides within the miR-22 binding site. **b** The relative luciferase activity in MHCC-97H and SMMC-7721 cells were determined after the CD147 3′UTR or mutant plasmids were co-transfected with miR-22 overexpression vector or negative control

To verify whether miR-22 directly targeted CD147 in HCC cell lines, luciferase reporter assays were conducted. We constructed pmirGLO-CD147-3′-UTR and pmirGLO-CD147-3′-UTR-mut with a substitution of four nucleotides within the miR-22 binding site (Fig. 5a). Cotransfection of MHCC-97H cells with pmirGLO-CD147-3′-UTR and miR-22/pcDNA3.1 caused a 59.3% decrease in the luciferase activity compared with the negative control (*P* < 0.05). This suppression was rescued by the four-nucleotide substitution in the core binding sites (Fig. 5b). The similar effect was also found in SMMC-7721 cells (68.3% decrease compared to the blank control, *P* < 0.05, Fig. 5b). These results indicated that miR-22 served as a tumor metastasis suppressor in HCC cell through the CD147 pathway.

## Discussion

Hepatocellular carcinoma is one of the most frequently occurring cancers with poor prognosis. Before the age of 60 years, HCC is the most commonly diagnosed cancer and the leading cause of cancer death in men [28]. But novel diagnostic or prognostic biomarkers and therapeutic targets for HCC are urgently required. With the advance of high-resolution microarrays and massively parallel sequencing technology, miRNAs are suggested to play critical roles in the tumorigenesis and development of human HCC.

miRNAs have been demonstrated to have close relationship with HCC. miR-22, originally identified in HeLa cells, has been found to be overexpressed in prostate cancer, but down-regulated in breast cancer, cholangiocarcinoma, multiple myeloma, and hepatocellular carcinoma [27]. miR-22 inhibits cell growth and induces cell-cycle arrest, apoptosis and senescence in breast cancer, colon cancer and lung cancer [29]. miR-22 plays a tumor-suppressive role by downregulating oncogenic target genes in many kinds of cancer [10, 24, 30, 31]. However, on the other side, miR-22 was recently suggested having an oncogenic role by targeting PTEN or TET family [8, 32]. The reported paradoxical functions of miR-22 imply that miR-22 might act as a tissue/cell-specific or context-dependent tumor suppressor microRNA and the function diversely depending on its target genes and related regulatory networks. Perhaps, miR-22 may play more complex roles that exceed our perception in cancer, which needs us to explore it more deeply.

In this study, we demonstrated that miR-22 expression was decreased in HCC tissues and cell lines compared detected by real– time RT– PCR. Furthermore, the expression of miR-22 in metastatic HCC tissues was much lower than in no metastatic HCC tissues which indicated that the miR-22 expression was correlated with the HCC metastatic ability. Overexpression of miR-22 in MHCC-97H and SMMC-7721 cells significantly inhibited cellular proliferation, migration and invasion capability in vitro. In the HCC tumor models, miR-22 expression vector decreased the HCC tumors growth. Taken together, our results suggest that miR-22 worked as a tumor suppressor miRNA and contributed to inhibit HCC cells migration and invasion in vitro.

Previous work showed that CD147 was more strongly upregulated in HCC specimens than in the adjacent tissues and that this overexpression correlated with tumor metastasis and advanced histologic grades [14, 33–35]. In the present study, we found an inverse correlation between miR-22 and CD147 expression in the HCC tissues. Our results are also shown that CD147 is negatively regulated by miR-22 at the posttranscriptional level, via a specific target site within the 3′UTR. miR-22 inhibits HCC cell migration and invasion through the CD147 pathway. Hence, our work indicates that miR-22 is an important suppressor in HCC invasion and metastasis, and CD147 seems to be a major downstream effector of miR-22 in its target network. Currently, the emergence of new technologies that use synthetic miRNA mimics or anti–miRNA oligonucleotides holds great promise for clinical miRNA therapy [36]. Synthetic miR-22 mimic treatments for cancer will become a significant scientific and therapeutic challenge.

In conclusion, miR-22 down-expressed in HCC and the overexpression of miR-22 inhibited cell migration and invasion of HCC cells in vitro, and decrease HCC tumor growth in vivo. CD147 contains the binding site for miR-22 and is negatively regulated by miR-22. Down-regulation of CD147 mediated miR-22 function. This newly identified miR-22/CD147 link provides a new, potential therapeutic target to treat HCC.

## Abbreviations

HCC: hepatocellular carcinoma
ANT: adjacent non-tumor
RT-PCR: reverse transcription polymerase chain reaction
MTT: 3-(4,5-dimethyl-2-thiazolyl)-2,5-diphenyl-2H-tetrazolium bromide
RNA: ribonucleic acid
mRNA: messenger RNA
GAPDH: glyceraldehyde-3-phosphate dehydrogenase
